# Knowledge on Nonpharmacological Methods of Pain Management among Nurses at Bindura Hospital, Zimbabwe

**DOI:** 10.1155/2019/2703579

**Published:** 2019-01-01

**Authors:** Estele Mwanza, Reginald Dennis Gwisai, Chiratidzo Munemo

**Affiliations:** ^1^Department of Health Sciences, Bindura University of Science Education, Zimbabwe; ^2^Department of Environmental Science, Bindura University of Science Education, Zimbabwe

## Abstract

This study is a quantitative descriptive study, which was conducted with an aim to assess the knowledge on nonpharmacological methods of pain management among registered general nurses at Bindura Hospital. This is because most nurses focus more on pharmacological pain management than nonpharmacological therapies which are given less attention or accord. This study used a descriptive study design, which is a nonexperimental research design so as to obtain information about registered nurses knowledge on nonpharmacological pain management. A convenience sampling technique was utilised to select a sample of seventy-five participants. Data were collected by the researcher who distributed self-administered questionnaires to available registered nurses after obtaining informed consent at Bindura Provincial Hospital. The mean knowledge score for this study was 48.6% and was below a pass mark of 50% and far below 80% which is the minimal acceptable level of knowledge on the Nurses Knowledge and Attitude Scale. A minimum knowledge score of 16% was obtained from participants showing lack of knowledge on indications of nonpharmacological pain therapies and a maximum knowledge score of 97.3% was shown on knowledge on nonpharmacological techniques. The following conclusion was drawn from the research findings; the study showed that the nurses have poor knowledge regarding nonpharmacological pain management as indicated by mean knowledge score of 48.6%. The researcher therefore recommends that the nursing practice should take an initiative in ensuring that all practicing nurses practice the highest possible pain management nursing care and that opportunities should be made available for nurses to be educated in effective pain management utilising nonpharmacological therapies.

## 1. Introduction

The concept of pain has been defined and explained from many dimensions. The International Association for the Study of Pain [1] defines pain as an unpleasant sensory and emotional experience associated with actual or potential tissue damage. Some studies state that pain is known as the fifth vital sign, and health professionals should monitor and manage it when caring for patients [2, 3]. There are three types of pain, based on where in the body the pain is felt, somatic, visceral, and neuropathic. As observed by previous studies [4], pain has physical harmful effects that may lead to physiologically unsafe conditions. Smeltzer* et al.*[5] also elaborate that inadequately treated pain has harmful effects such as sleep alterations [6]. Of particular importance to nursing care, unrelieved pain reduces patient mobility, resulting in complications such as deep vein thrombosis, pulmonary embolus, and pneumonia [4, 7]. Bernhofer [8] also states that undertreated pain leads to respiratory, cardiac and endocrine complications, and delay in healing and potentiates the onset of chronic pain. Despite the growing awareness on pain management, patients still suffer from unnecessary pain in many hospitals with the resultant negative effects on physical, emotional, and spiritual health and quality of life [9–11]. Pain management is an important aspect of patient care and nurses play a significant role in the acute care setting in providing pain assessment and treatment [9, 12]. The use of nonpharmacological pain relief techniques has been found to be effective with less side effects and complications associated with them (Rakel and Barr, 2010). On a global perspective, the prevailing persistent challenge in the use of nonpharmacological pain techniques has been expressed in many studies as care provider attitude and lack of knowledge [1]. Previous studies reveal that nondrug methods of pain management do the following, diminish pain perception by reducing intensity and increasing pain tolerance, reduce pain-related distress, strengthen coping abilities; and give the patient and family a sense of control over pain [6, 13]. Traditionally, pain management tends to emphasize the use of pharmacological agents. However, pain is influenced by an array of physical and psychosocial factors, and patients differ in their response to pain and to analgesics. Therefore, it is important to have a range of options, including nonpharmacological therapies available, in order to manage patients' pain effectively. Evidence in the African context shows that pain has been under managed [14]. In Zimbabwe, previous studies do not show many discrepancies in the use of the nonpharmacological pain control methods, with knowledge and attitudes playing a critical factor in terms of practice [6, 7]. Kipkoriri [15] reveals that the majority of women in labour who used nonpharmacological techniques reported control of pain. However uptake of these complementary methods of pain management is still low by both clients and health workers in Zimbabwe [15]. This study is aimed at improving the quality of patient care, providing the nursing fraternity with bases of research areas, widening the scope of nursing practice, and adding nonpharmacological remedies to the registered nurse curriculum [2]. Furthermore, nonpharmacological pain management has not been considered as an option, such that the nursing curriculum has not enucleated it as a subject on its own [6, 7, 16]. This study will assist in improving the acceptance levels and inclusion of nonpharmacological therapies in basic nursing programmes as a topic or subject area to be studied extensively [2]. On the other hand, lack of knowledge together with other barriers to implementation of these therapies has let patients suffer inadequate pain relief [9, 17]. Overall, there has been little utilisation of nonpharmacological pain management modalities [7].

## 2. Materials and Methods

### 2.1. Description of Study Area

**Figure 1:**
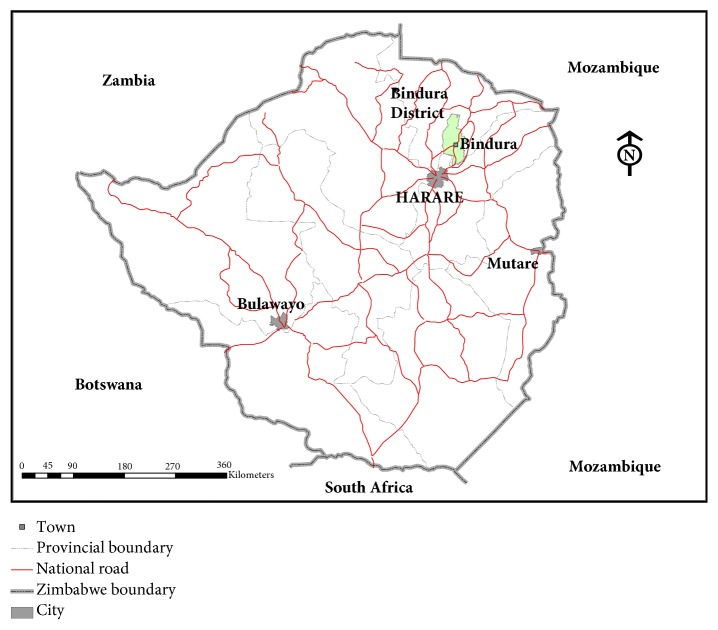
Map of study area.

Bindura Provincial Hospital is a government hospital in Bindura town, in Mashonaland Central Province of Zimbabwe. It is about ninety kilometres away from Harare the capital city of Zimbabwe (Figure 1). It serves as a secondary level health delivery system and has a total of one hundred and twenty-five registered nurses as at the year ending 2015. The hospital has the following departments: casualty, outpatient, male ward, female ward, intensive care unit, maternity, operating theatre, family and child health, dental, eye unit, and a children's ward. The hospital offers preventive, curative, and rehabilitation clinical services for outpatients and in patients. The site was also chosen because it is the largest hospital in the area which admits patients within Bindura as well as surrounding towns; thus nurses at the hospital will have had an experience in nursing medical admitted adult patients. The hospital was also chosen due to the possibilities of getting a larger sample population for the study.

## 3. Map of Study Area

### 3.1. Sampling Method

Only registered nurses who work in the following departments were eligible to participate in the study: outpatient, male ward, female ward, maternity, eye unit, and family and child health. Furthermore, nurses on duty in the selected wards or departments who fell within the selected sample drawn on the specified day of data collection and consented were eligible for participation. The data was collected from seventy-five registered nurses through the use of self-administered questionnaires and informed consent was obtained from all participants. Participant's anonymity was promoted by issuing self-administered questionnaires in the selected departments.

## 4. Results

### 4.1. Demographics

Table 1 show the highest number of respondents to be in the age group 28 to 37 category (62.7%). The majority of the respondents were females (74.7%). On the other hand most (58.7%) of nurses had a general nurse diploma as their highest qualification. Based on experience in years the highest number of respondents (21.3%) was in the below 5 years category and the majority of the respondents were Christians (98.7%).

**Figure 2:**
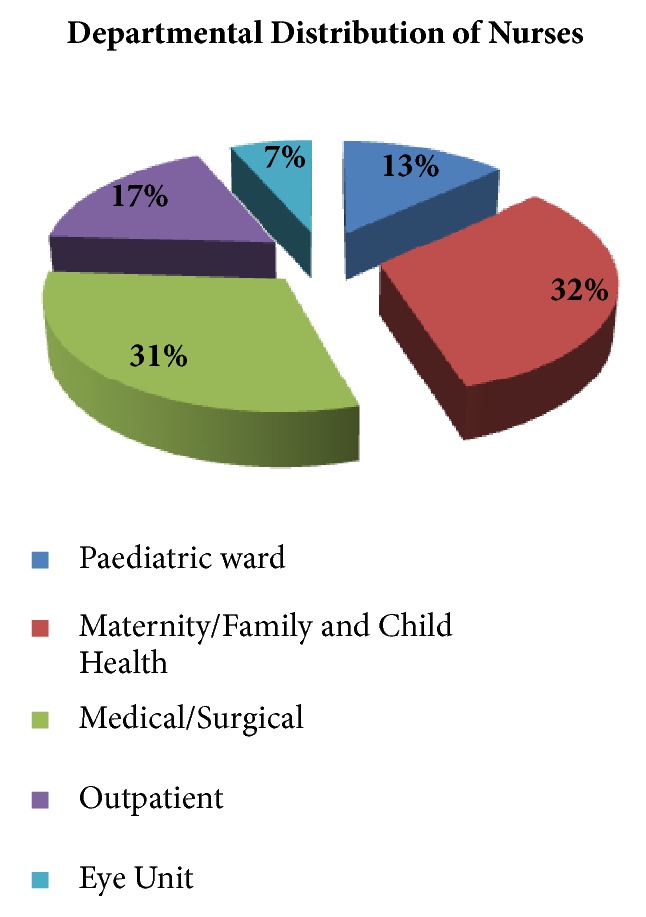
Distribution of respondents by departments (*n=75*).

The highest number of participants (registered nurses) came from the maternity, family, and child health department (32%), followed by the medical and surgical department (31%) (Figure 2).

### 4.2. Knowledge on Nonpharmacological Pain Management

Table 2 shows that most respondents had knowledge on the following: imagery (93.3%), heat and cold therapies (93.3%), Transcutaneous Nerve Simulation (TNS), (76%), Acupuncture (69.3%), and relaxation (97.3%). Furthermore, the majority felt nonpharmacological therapies have no side effects (56%), cannot be replaced (56%), have different modes of action (62.6%), and relieve pain by altering pain perception (73.3%). On the other hand, based on relaxation techniques, most respondents felt that nonpharmacological therapies decrease respiration (50.7%) and muscle spasms (72%), despite an increase in heart rates (42.7%).

**Figure 3:**
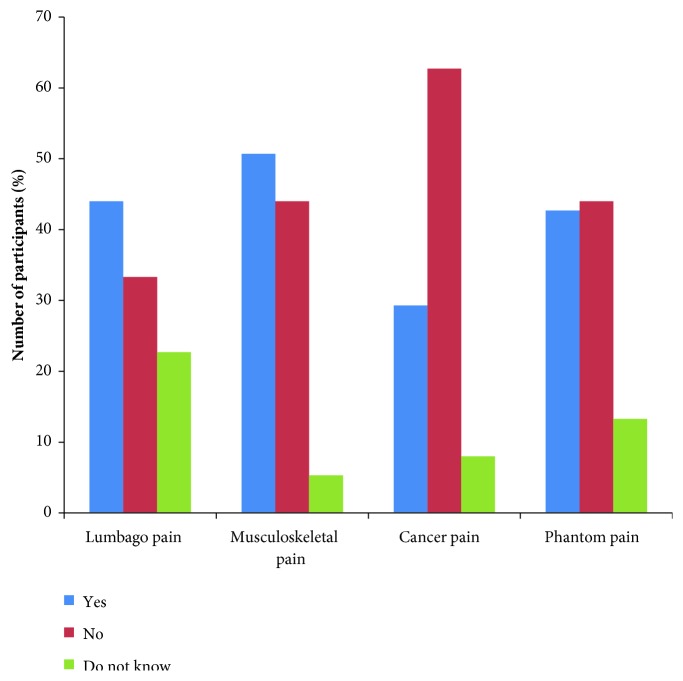
Knowledge on type of pain where nonpharmacological pain therapies are applicable (*n=75*).

**Figure 4:**
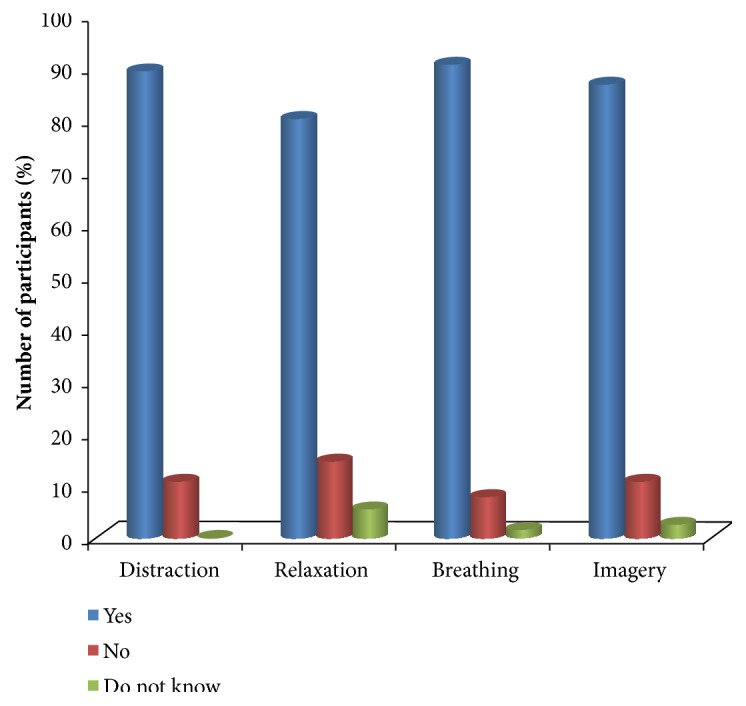
Knowledge on nonpharmacological techniques (*n=75*).

The majority of the respondents accepted the use of nonpharmacological therapies for treating musculoskeletal pain (50.7%), followed by Lumbago pain (44%) as compared to other forms of pain (Figure 3). Furthermore, most respondents identified distraction (89.3%), relaxation (80%), breathing (90.7%), and imagery (86.7%) as techniques of reducing pain (Figure 4).

Table 3 shows that most participants were in agreement with the following notions: nurses need to undergo special training on nonpharmacological management (60%), and nonpharmacological management may be used as an alternative to pharmacological pain management (56%). Furthermore, considering the resumption of nonpharmacological management, the most respondents were positive to start when patients gained control of pain (68%) and agreed to begin nonpharmacological management as soon as pain is reported (68%). On the other hand, most participants did not agree to begin nonpharmacological management after completion of drugs (54.7%). The majority, agreed to begin nonpharmacological management between doses of pain medication (60%), while others felt that nonpharmacological pain management could be commenced simultaneously with pain medication (58.7%).

**Figure 5:**
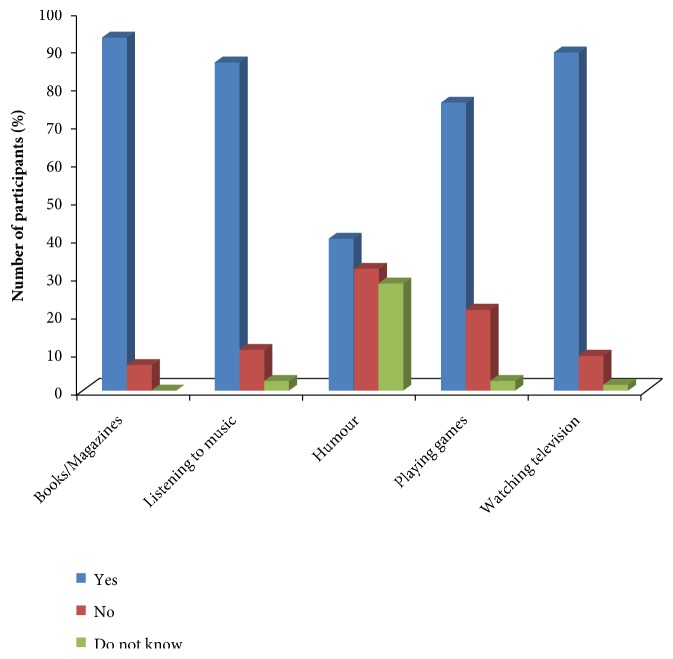
Knowledge of diversion techniques (*n=75*).

Figure 5 shows respondents' knowledge on forms of diversion techniques, where most participants supported the use of magazines (93.3%), music (86.7%), playing games (76.1%), and watching television or videos (89.3%).

Table 4 shows the yes, no, and do not know frequency from participants pertaining various questions based on who should apply nonpharmacological therapies. Most respondents (64%) agreed that all members of the pain team can recommend use of nonpharmacological therapies at acute phases of injuries (70.7%); nonpharmacological therapies can be learnt and applied by nurses (84%). On the other hand, most respondents did not recommend the use of nonpharmacological management postoperative pain (72%), did not recommend use on paediatric pain (66.7%), disagreed with the use of nonpharmacological management on neuralgic pain (73%), and did not recommend its use for cancer pain (78.7%).

## 5. Discussions

### 5.1. Knowledge on Nonpharmacological Therapies

A central finding in this study was that the nurses were knowledgeable about nonpharmacological approaches in pain management. The majority of the respondents managed to identify imagery, heat, and cold therapies as types of nonpharmacological therapies. This was similar to previous studies conducted elsewhere [18]. Furthermore, most respondents reported that Transcutaneous Nerve Stimulation (TNS) is a nonpharmacological pain therapy, and this was much higher than observations in previous studies [3]. Another observation was that an overwhelming majority of respondents reported that relaxation is a nonpharmacological pain therapy and this was consistent with previous studies conducted elsewhere [3].

The majority of respondents showed knowledge on nonpharmacological pain management by stating that nonpharmacological therapies do not replace pharmacological therapy whereas other studies revealed a contrasting view [19]. Most respondents showed lack of knowledge on palliation. This is similar to reports of previous studies [20]. Furthermore, most respondents reported that nonpharmacological pain therapies have different modes of action. This is in contrast with previous studies where a limited percentage had knowledge on how nonpharmacological pain therapies work [21]. Most respondents affirmed that nonpharmacological pain therapies relieve pain by altering pain perception, a result lower than that reported by other studies [22]. A significant figure stated that relaxation techniques increase the heart rate a notion in contrast to other studies that have shown a decrease in heart rates through relaxation techniques [22]. This shows that most respondents lack knowledge on this aspect of nonpharmacological pain therapy. The majority of respondents reported that relaxation techniques decreased respiration rate, blood pressure, and muscle spasms which were similar to previous studies [23, 24].

Most respondents identified distraction as a technique of reducing pain which was also observed in other studies where respondents reported distraction as a pain reducing technique [20]. Also, the majority of the respondents reported relaxation to be a pain reducing technique which was consistent with the findings of previous studies [25]. This study found out breathing and imagery to be pain reducing techniques. This is almost similar to results obtained by other scholars where respondents identified breathing and imagery as nonpharmacological pain methods [26]. Furthermore, Barrett [27] observed that nurses identified distraction, relaxation, and imagery as nonpharmacological pain therapy.

A small proportion of respondents reported that special training is required for nurses to administer nonpharmacological techniques. However the majority of respondents showed lack of knowledge on nonpharmacological management as an alternative to pharmacological pain management. This was comparable to results that were reported by Chou and Shekelle [28], who indicated that most respondents lacked adequate knowledge on nonpharmacological pain therapy as an alternative to pharmacological pain management. Most respondents reported that the best time to start nonpharmacological management was when patients gained control of pain which was in contrast to previous studies conducted elsewhere [29]. The majority agreed to start nonpharmacological management as soon as pain was reported which is consistent with previous studies conducted [30]. On the other hand a majority of the respondents disagreed that nonpharmacological management is started after completion of drugs, while most respondents reported that nonpharmacological pain management should be started between doses of pain medication, a figure higher than that reported by other studies elsewhere [3]. The majority of respondents reported that nonpharmacological pain management may be commenced simultaneously with pain medication, which showed a fair level of knowledge. The result is comparable to that obtained by other studies conducted [3].

On diversion techniques, the majority rated the following as diversion: magazines, music, and humour, respectively. The findings are almost similar to those reported by other studies [31]. Furthermore, the majority of respondents accepted playing games as diversion, whereas other studies reported a lower percentage of respondents with the same viewpoint [31]. A higher score was obtained among respondents on watching television/videos as a diversion therapy, a result consistent with other studies conducted elsewhere [22]. The majority of the respondents reported that all members of the pain team could apply nonpharmacological therapies. This is in agreement with what was reported by previous studies [20]. Furthermore, most respondents believed that nonpharmacological therapies could be learnt and applied by nurses, and similar findings have been observed to that effect [20]. A small proportion recommended nonpharmacological therapies in acute phases of injury while the majority of respondents would not recommend in acute phase. This is however in contrast with other reports [30].

## 6. Conclusion

The study showed that the nurses have poor knowledge regarding nonpharmacological pain management as indicated by the mean knowledge score of 48.6% against a pass mark of 50% and a recommended knowledge score of 80% as observed by previous studies and other yardsticks [32].

## 7. Recommendations

From the results of the study, the study recommends that nurses should engage in continuous professional development and continued education, by studying recent research on pain management and practicing evidence based skills learnt so as to improve pain management nursing care. The nursing education curriculum should place more emphasis on nonpharmacological pain therapies in order to equate pharmacological therapy. Furthermore, the study recommends that the nursing practice should take an initiative in ensuring that all practicing nurses should offer the highest possible pain management nursing care and that effective training should be conducted on effective pain management utilising nonpharmacological therapies. Also, a study research using large samples and comparative cohorts is recommended for Zimbabwe to upscale and enhance the generalizability of the findings and to improve the nursing knowledge base.

## Figures and Tables

**Table 1:** Demographic profile (*n=75*).

**Variable**	**Category**	**Frequency**	**Percentage**
Age	18 to 27	3	4
	28 to 37	47	62.7
	38 to 47	19	25.3
	48 +	6	8
Sex	Female	56	74.7
	Male	19	25.3
Academic qualification	General Nurse Diploma	44	58.7
	BSc. Nursing Degree	5	6.6
	Any other	26	34.7
Experience in years	Below 5 years	16	21.3
	6 to 10 years	43	57.3
	11 to 15 years	14	18.6
	16 to 20 years	2	2.7
	Above 21 years	0	0
Religion	Christianity	74	98.7
	Muslim	1	1.3
	Traditionalism	0	0
	Any other	0	0

**Table 2:** Knowledge on nonpharmacological pain management techniques (*n=75*).

**Variable**	**YES**		**NO**		**DO NOT KNOW**	
	Frequency	%	Frequency	%	Frequency	%
**The following are nonpharmacological therapies**						
Imagery	70	93.3	4	5.4	1	1.3
Heat and cold therapies	70	93.3	5	6.7	0	0
Transcutaneous electrical nerve stimulation	57	76	12	16	6	8
Acupuncture	52	69.3	14	18.7	9	12
Relaxation	73	97.3	0	0	2	2.7
**Pain Therapies**						
Contain no side effects	42	56	20	26.7	13	17.3
Do not replace pharmacological therapy	42	56	25	33.3	8	10.7
Can be used as palliation	19	25.3	35	46.7	21	28
Have different modes of action	47	62.6	8	10.7	20	26.7
Relieves pain by altering pain perception	55	73.3	8	10.7	12	16
**With Relaxation Techniques**						
Heart rate increases	32	42.7	33	44	10	13.3
Respirations decreases	38	50.7	23	30.7	14	18.6
Blood pressure increases	19	25.3	45	60	11	14.7
Muscle spasms decreases	54	72	10	13.3	11	14.7

**Table 3:** Knowledge of nonpharmacological pain management (*n=75*).

**VARIABLE**	**YES**		**NO**		**DO NOT KNOW**	
	Frequency	%	Frequency	%	Frequency	%
Nonpharmacological pain management require nurses to undergo special training	28	37.4	45	60	2	2.6
Nonpharmacological pain management can be used as an alternative to pharmacological therapy	42	56	33	44	0	0
When the patient has gained control of the pain	48	64	23	30.7	4	5.3
As soon as pain is reported	51	68	24	32	0	0
After completion of pain medication	33	44	41	54.7	1	1.3
Between doses of pain medication	45	60	23	30.7	7	9.3
Simultaneously with pain medication	44	58.7	31	41.3	0	0

**Table 4:** Knowledge on nonpharmacological therapies (*n=75*).

**VARIABLE**	**YES**		**NO**		**DO NOT KNOW**	
	Frequency	%	Frequency	%	Frequency	%
Nonpharmacological pain therapies can generally be applied by all members of pain team	48	64	22	29.3	5	6.7
Nonpharmacological pain techniques can be learnt and supplied by nurses	63	84	9	12	3	4
**Would you recommend non pharmacological pain therapies**						
In the acute phase of injury	22	29.3	53	70.7	0	0
In post-operative pain management	19	25.3	54	70.1	2	2.6
On a paediatric patient in pain	25	33.3	50	66.7	0	0
Neuralgic pain	12	16	55	73.3	8	10.7
Cancer pain	14	18.7	59	78.7	2	2.6

## Data Availability

The data used to support the findings of this study are available from the corresponding author upon request.
